# Membrane Bioreactors for Produced Water Treatment: A Mini-Review

**DOI:** 10.3390/membranes12030275

**Published:** 2022-02-27

**Authors:** Dennis Asante-Sackey, Sudesh Rathilal, Emmanuel Kweinor Tetteh, Edward Kwaku Armah

**Affiliations:** 1 Green Engineering and Sustainability Research Group, Department of Chemical Engineering, Faculty of Engineering and the Built Environment, Durban University of Technology, Durban 4001, South Africa or dennis.asantesackey@kstu.edu.gh (D.A.-S.); rathilals@dut.ac.za (S.R.); or ekarmah@cktutas.edu.gh (E.K.A.); 2 Department of Chemical Engineering, Faculty of Engineering and Technology, Kumasi Technical University, Kumasi P.O. Box 854, Ghana; 3 Department of Applied Chemistry, School of Chemical and Biochemical Sciences, C.K. Tedam University of Technology and Applied Sciences, Navrongo P.O. Box 24, Ghana

**Keywords:** fouling, membrane bioreactors, oil and gas wastewater, produced water

## Abstract

Environmentalists are prioritizing reuse, recycling, and recovery systems to meet rising water demand. Diving into produced water treatment to enable compliance by the petroleum industry to meet discharge limits has increased research into advanced treatment technologies. The integration of biological degradation of pollutants and membrane separation has been recognized as a versatile technology in dealing with produced water with strength of salts, minerals, and oils being produced during crude refining operation. This review article presents highlights on produced water, fundamental principles of membrane bioreactors (MBRs), advantages of MBRs over conventional technologies, and research progress in the application of MBRs in treating produced water. Having limited literature that specifically addresses MBRs for PW treatment, this review also attempts to elucidate the treatment efficiency of MBRs PW treatment, integrated MBR systems, general fouling, and fouling mitigation strategies.

## 1. Introduction

The petroleum refinery industry plays a major role in providing energy to meet the world’s energy demand and industrial activities; its exploration comes with huge environmental risks. Despite the rate of renewable energy growth due to fossil fuel depletion, the oil and gas industry is anticipated to contribute 40–50% of the total global energy mix between 2040–2050 [1,2]. The value chain of crude oil and gas can be categorized into the upstream (exploration, drilling, and extraction), midstream (processing, transportation, and storage) and downstream (conversion, refinery, marketing and distribution) [3,4], where the transportation in the midstream is mostly via pipeline, rail, and shipments. 

Consequentially, refining crude oil into useful products demands huge amounts of water for processes such as distillation, cracking, polymerization, alkylation, hydrotreating, desalting, treatment, and finishing of petroleum products [3,5,6]. In this case, it is estimated that one barrel of refined oil produces nearly 10 barrels of wastewater [5]. This wastewater is highly complex, containing high concentrations of residual free and emulsified oils, hydrocarbons (representing the majority of the organic load), dissolved salts (halides, phosphates, sulfates, and sulfides), and carcinogenic and mutagenic substances that require rigorous intervention to be eliminated [5,6,7,8]. During oil and gas processes, about 80% liquid waste, often at temperatures >50 °C is commonly referred to as produced water (PW) [7]. The ratio of PW to oil extracted from the reservoirs are estimated at 3:1 [6,7]. Basically, the total water consists of water in the cavities of the subsurface formations and injected water which has a dual purpose of enhancing the recovery of oil and gas and maintaining pressure in the reservoir [9,10,11]. For gas production wells, PW also consists of condensed water [12,13]. Inclusive of water, PW is also a complex mixture of soluble and insoluble high molecular weight hydrocarbons (aromatic and saturated), heavy metals, anions, and other impurities [14]. Their varying quantity, constituents, and characteristics are dependent on the geographic location of the field, geo-structure of the well, age of well, reservoir drive mechanism, mechanical integrity, well drilling and production technology, refinery technology, and the type of hydrocarbon products [15,16].

Water demand and issues of scarcity cannot be overlooked as water is essential to life and the functioning of ecosystems on earth. The demand and withdrawal concerns does not pertain to only volume, but rather, to volume and quality [17]. A fundamental proceeding in the protection of quality water involves investigating and developing efficient technologies for the treatment and conversion of these complex toxic compounds into harmless ones. A viable route to water sustainability and fresh water scarcity is the recycling and reuse of reclaimed wastewater for non-potable and potable use [18,19]. This environmentally and financially sustainable approach also involves the recovery of resources such as nitrogen, organic matter, phosphorus, heavy metals, and value addition of sludge through its beneficial usage as fertilizers, green energy, and biosolids [20]. 

For the beneficial reuse of PW for agricultural irrigation, a much-supported application that is aimed at transforming waste streams into a valuable resource requires a level of treatment before using to avoid operational challenges [21,22]. In the treatment of PW, the general objectives are to achieve the removal of both free and dispersed oil (de-oiling), disinfection, the removal of suspended particles, removal of various dissolved gases, desalination, and demineralization, softening and deionization, and sodicity level adjustment. Comparing membrane bioreactors (MBRs) to Physico-chemical processes, trickling bed, and activated sludge, MBRs have shown quite high treatment performances in removing both organic and inorganic contaminants [23,24,25]. While most of the reviews available consider generalized performances of membrane bioreactors on generalized wastewater, less attention has been paid to MBRs for PW treatment. This review, based on a systematic literature survey approach, considers the application of MBRs specifically to PW treatment. Sections of this succinct, easy to refer review expounds on PWs, MBRs, and the output of the investigations on MBRs in PW treatment, which includes the system configuration, integration of MBR, and other processes to treat PWs and modeling of MBR in PW treatment. The remaining phase is an overview of fouling in MBRs in PW treatment and fouling control in MBRs with its adaptability in PW treatment. Finally, the authors provide their perspective on the future of MBR research in PW treatment, which is expected to provide research gaps to scientists and engineers engaged in this field.

## 2. Produced Water

### 2.1. Production and Mangement of PW

**Figure 1:**
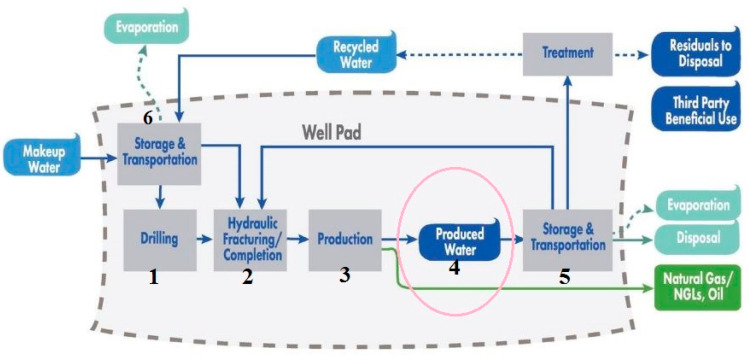
Water life cycle of PW in an unconventional oil and gas production [28].

Globally, PW generation is estimated at 250 million barrels per day with a foreseeable increase that is aligned to the increased oil and gas production and ageing of wells [16]. PW from oil and gas exploration fields contribute to 60% of annual generation. About 30% of the global oil production is contributed by offshore production [26]. In the United States of America (USA) alone, an estimated 21.6 billion barrels of PW is generated every year with onshore production contributing to about 97% and the remainder attributed to offshore sites [27]. PW from offshore exploration is mostly discharged to the immediate aquatic environment. Figure 1 shows the water life cycle of PW. However due to the hazardous constituents of PW, its management has been met with stricter oil and gas policies and legislation to avoid interference with environmental sustainability. These regulatory policies and standards vary from country to country and non-compliance could result in civil penalties, large fines, international criminal prosecution, and lost or deferred production [28]. One major concern of relevance that has led to these legal considerations is the biological effect of PW. Adopted management practices include the reuse of PW for drilling and operational purposes and deep well injection with no intent of accessibility [29]. The most adopted energy demanding, carbon-intensive, and expensive injection technique costs between $0.3–10 USD per barrel with associated environmental effects such as underground water contamination and induced seismicity [30,31,32]. 

### 2.2. Characteristics of Produced Water

PW is hypersaline in nature and it is, therefore, denser than seawater. Compared to the 30 g/L of total dissolved solids (TDS) of sea water, as high as 300–400 g/L has been recorded for PW from some oil and gas fields [33,34,35]. Hypersaline concentration up to 800 g/L has previously been reported [36]. The dissolved Na^+^ and Cl^−^ contributes more to salinity than the chloride salts of calcium, magnesium, and potassium. Findings show that the higher the temperature of the reservoir, the higher the TDS concentration in PW [37]. Aside chloride ions, other anions present in PW includes sulfate, carbonate, and biocarbonate. As such, much of this environmental concern arises when the PW is discharged into land surface and fresh water rather than the ocean [38,39]. 

The range of chemical additives used in drilling exploitation and production are not limited to corrosion and scaling inhibitors, emulsion breakers, fracturing fluids, clarifiers, solvents, coagulants, surfactants, biocides, and flocculants [40,41,42]. These treatment chemicals at a lower concentration of 0.1 ppm are considered highly toxic. This array forms part of the molecular composition of the PW. During the oil and gas extraction process, technologically enhanced naturally occurring radioactive materials (TERNOMS) or naturally occurring radioactive materials (NORM) are found in drill cuttings, flowback water, pipe scale, sludges, sediments, and filters captured as liquid or gases [43]. 

A quick comparison on the bases of extraction fields reveal that PW from gas fields is lower in volume but has high acidity and higher concentration of volatile components. The pH of PW from oil fields fall on a wide scale of 4.3–10 while that of gas fields range between pH 3.10–4.4. Additionally, benzene, toluene, ethyl benzene, xylene (BTEX), and naphthalene are found in higher concentrations in PW from gas fields than PW from the oil fields [44]. Straight chain alkanes (C_10_–C_30_) are the most dominant hydrocarbons, approximated at 90% in detection of which 25% is higher molecular weight n-alkanes ranging from C_21_ to C_34_. Table 1 shows a list of other constituents in PW. With petroleum compounds making up PW constituents, total organic carbon (TOC) of PW is also expected to be high. 

### 2.3. PW Treatment Technologies

In general, wastewater treatment technologies can be categorized into preliminary, primary, secondary, and tertiary treatments. Preliminary treatment involves separating of entrained coarse solids such as sticks, grits, rags, and other floatable suspended solids. The primary process uses filtration and sedimentation to remove portions of suspended and organic matter, thereby accomplishing about 50–70% suspended solids and 35–40% biochemical oxygen demand [65,66,67]. Secondary treatment incorporates biological processes such as activated sludge and trickling filtration together with chemical precipitation in achieving high effluent. An estimated 85–95% biological oxygen demand (BOD) and suspended solids removal can be accomplished for a well operated and designed secondary treatment plant [68,69]. Also referred to as the polishing stage, tertiary treatment is required as an add-up when effluent characteristics from the secondary treatment process does not meet regulatory requirement. An estimated 99% removal is achieved in this process and it may involve physico-chemical techniques [70,71]. 

Various physico-chemical and biological treatments widely known in wastewater treatment have been applied to produce water are alkaline chemical precipitation, adsorption, ion exchange, membrane separation, and coagulation-flocculation [72,73]. Technologies—such as hydrocyclone, gas flotation, and gravity-based separators have also been used in the oil and gas sector for water purification and the reduction of oil–water concentration [74]. Table 2 accounts for the advantages and disadvantages of some available PW treatment technologies. 

## 3. Membrane Bioreactors (MBRs)

MBRs, a combination of selective membrane process such as microfiltration or ultrafiltration and a biological process in a simplified single unit has become an alternative technology for wastewater treatment. Being an alternative to the conventional activated sludge (CAS), MBRs have attracted much attention as a cost competitive and effective alternative. Whilst the biological process of the MBR degrades organic pollutants by adapted microorganisms, the membrane establishes a physical barrier that separates the biomass from the treated wastewater [82], hence, the possibility of recycling the activated sludge as recycled activated sludge (RAS). 

**Figure 2:**
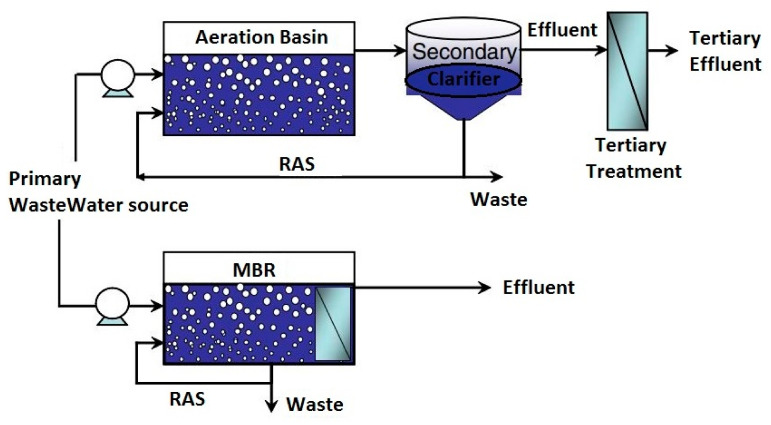
Difference between CAS and MBR. Adapted from [87].

The introduction of membrane bioreactors (MBRs) as a treatment technology has given rise to reduced plant foot print through the elimination of processes such as secondary clarification, tertiary filtration and UV disinfection and works with low feed to microorganism ratio [83,84]. Other advantages of MBRs include good disinfection capability, high quality effluent, good organic and inorganic separation ability, high organic loading resisting capability, absolute control for a longer sludge retention time (SRT) and shorter hydraulic retention time (HRT), and low sludge production rate due to negligible settleability [85,86,87]. Having a longer SRT allows slow-growing microorganisms responsible for the degradation of most nitrogen-based compounds to develop. Over the years, MBRs have been used to effectively treat industrial and municipal wastewater at mixed liquor suspended solids (MLSS) levels up to 12 mg/L, a level that CAS can only handle a minor fraction [88]. Despite treatment reliability of MBRs, they are associated with high operational and capital costs, membrane fouling phenomena, and high energy demand [89]. A visual comparison of CAS and MBR is shown in Figure 2.

**Figure 3:**
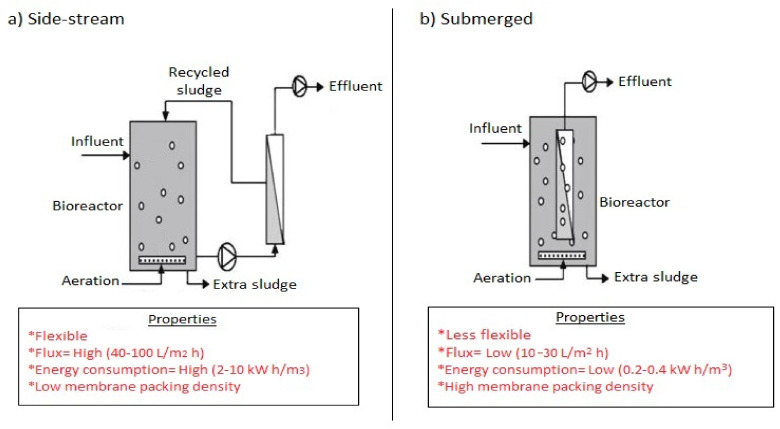
Schematic diagram of (**a**) side stream MBR and (**b**) submerged MBR.

Over 50 years from the first CAS patent [90], history has it that, Smith et al. [91] in 1969, under the Dorr-Oliver research program introduced the MBR technology. In place of a sedimentation tank used in a typical CAS system, Smith et al. [91] installed an ultrafiltration membrane outside the bioreactor. Despite high-quality effluent produced out of the treatment of sewage, energy consumption from the recirculating pump, membrane fouling, and specific applicability restricted its wide usage in North America. However, a configurational improvement and innovation from the pioneering work of Yamamoto et al. in 1989 included the placement of membranes into the bioreactor unit, installation of pressured pumps to circulate mixed liquor and application of suction pressure into the unit [92]. These earlier designed configurations now exist as first generation side-stream MBRs where the activated sludge flows at high velocities through a tubular or flat sheet membrane module in a typical cross flow filtration mode and the second generation MBRs where the membranes are submerged in the aerated tank in a more dead end filtration mode [93]. The energy consumption in the side stream is therefore usually higher due to the recycle flow velocity. Figure 3 shows the two configurations for (a) side stream and (b) submerged configurations. Over the years, modifications have resulted in the airlift external circulation (AEC) MBR with a combined advantage of the side-stream and submerged MBRs. Additionally, air sparging control for hollow tube membranes used in side streams and a solid retention control (SRC) system has been developed as improvement to the conventional MBRs.

In MBRs, the microfiltration (MF) and ultrafiltration (UF) membranes are primarily used with UF being the most effective for oily wastewater treatment when compared to the low efficiency and high operational cost of conventional methods [94]. The MF and UF membranes utilize a separation mechanism to retain micron and macromolecular or particles specifically in the range of 0.1–10 μm for MFs and 5 to 100 nm for UFs. Therefore, considering the functionality of the membrane in the MBR system, the general mass balance for solute separation in the process can simply be presented as

(1) $$Q_{f}C_{f}=Q_{p}C_{p}+Q_{C}C_{c}$$

where *Q* = flowrate, *C* = solute concentration, and subscripts *f*, *p*, and *c* denote the feed stream, permeate stream, and concentrate stream, respectively. 

The materials used in making these membranes can be grouped into ceramic, polymeric, and composite or modified membranes. Although ceramic membranes have excellent fouling resistance, chemical and mechanical stability, and low operating costs, their high manufacturing and brittle nature makes polymeric membranes a popular choice for MBRs [95,96]. However, due to the hydrophobic nature, polymeric membranes are easily fouled. Polymeric membrane materials include polytetrafluoroethylene (PTFE), polyvinylidene fluoride (PVDF), polysulfone (PSO), polyacrylonitrile (PAN), polyethylene (PE), polypropylene (PF), polyvinyl butyral (PVB), and polyethersulfone (PESO) [66]. A composite or modified membrane is a combination of different materials; one as active surface and the other as a layer support to provide a synergic effect. Typical to surface modification is using plasma treatment and incorporation of photocatalytic nanomaterials. 

**Figure 4:**
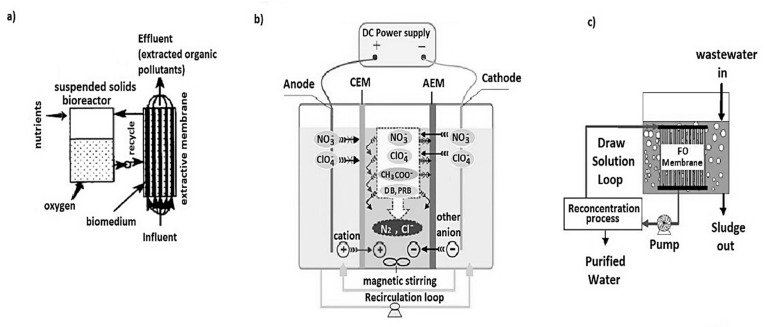
(**a**) Extractive membrane reactor; (**b**) Electro-dialysis ion exchange membrane bioreactor; (**c**) Osmotic bioreactor adapted from [109,110].

Based on the purpose of the membrane usage, MBRs are divided into four categories namely, (1) biomass separation membrane bioreactor (BSMBRs), (2) membrane aeration bioreactors (MABRs), (3) extractive membrane bioreactor (EMBR) and (4) ion exchange membrane bioreactors (IEMBRs) [97]. The BSMBRs serve as biomass separators in wastewater settings. In MABRs, the membrane cavity is supplied with pressurized oxygen or air which diffuses though the membrane pores [98]. The biofilm on the membrane side receives the oxygen, creating a nutrient rich profile for better removal of the pollutants as the counter diffusion of the bubbleless aerating oxygen and substrate occurs [99,100]. On the side of EMBRs, hydrophobic-organophilic membranes are used to provide selective transport of specific toxic recalcitrant organic compounds such as dichloroaniline, chloronitrobenzene, phenol, nitrates, and many more through the solution-diffusion mechanism into biofilms for biodegradation as the wastewater passes through the membrane lumen [101,102,103,104]. The IEMBR was patented two decades ago and it incorporates Donnan dialysis, a concentration gradient driven counter transport process where the feed stream with targeted ionic pollutants pass through a non-porous anion exchange membrane into a receiving bio-compartment, which bio reduces under anoxic conditions [105,106,107,108]. This includes the electrodialysis ion exchange membrane bioreactor (EDIMB) and the innovative osmotic membrane bioreactor (OsMBR) which has also been investigated independently on a laboratory scale [109,110]. Schematic diagrams of an EMBR, EDIMB, and OsMBR are presented in Figure 4. 

### 3.1. MBR in PW Treatment

**Figure 5:**
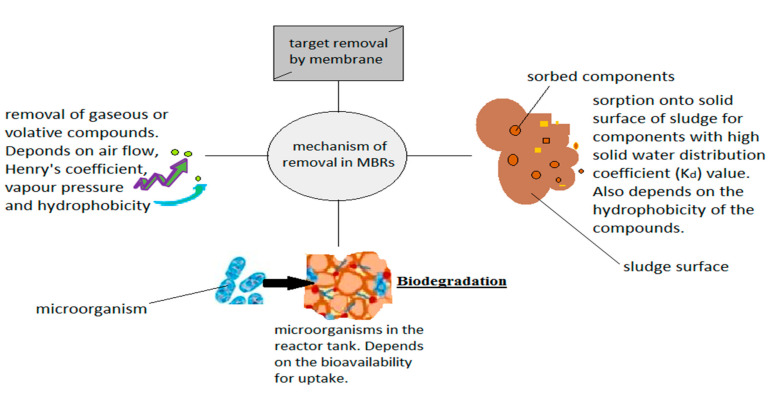
Treatment mechanism of an MBR.

This subsection tabulates the various experimental works performed using MBRs in PW treatment which takes into account the MBR performances. A summary of the mechanism of action is shown in Figure 5 while the treatment performance of MBRs, mostly dominated by the submerged MBR is shown in Table 3. From Table 3, it is established that MBRs are efficient in treating PW pollutants having observed an 80 to >90% oil and grease removal, COD (>90%), TOC (>90%), and 30 to > 60% for phosphate.

#### 3.1.1. Integrated Treatment Processes

Integrated treatment processes in the wastewater treatment industry have been widely practiced to obtain treatment wastewater that meets the legislated quality standards. This involves the combination of conventional and hybrid systems which can be categorized as the fusion of physical, biological and chemical processes. The synergic goals are not limited to overcoming the limitations in a stand-alone process. Notable among integrated processes for treating PW are connected process streams of sedimentation, hydrocyclone, electrocoagulation, reverse osmosis [117], aeration skimmer, and activated sludge-filtration [44], a PW zero discharge system consisting of coagulation, flocculation, filtration (sand), adsorption (granulated activated carbon), RO and crystallization [118], Ti/IrO_2_–Ta_2_O_5_, and BDD electrodes for a flotation and photo-Fenton technique [119], an integrated biochemical and capacitive deionization system called microbial capacitive desalination cell (MCDC) [120], gravity separation-hydrocyclone-sand filtration for non-H_2_S PW and gravity separation-induced gas flotation-nutsell filtration for PW containing H_2_S [121] and an infused pretreatment process consisting of gravity driven ultrafiltration, solar aeration, and GAC adsorption [122]. The gravity separation-hydrocyclone-nutshell filtration-mechanical vapor compression-storage chain process was also considered for the treatment of PW for internal reuse. Additionally, integrated pressure systems for desalination that combines reverse osmosis and chemical or slurry precipitation has been used in the treatment of produced water due to the salt concentration range [123]. 

Although there is limited literature on integrated systems involving MBRs, the organic removal and total membrane resistance of an SMBR and an integrated system consisting of ozonation and a moving bed biofilm SMBR has recently been compared. Lui et al. [124] reported that the total membrane resistance was 40.1% lesser in the integrated system with removal rates of DOC and total nitrogen reported as 3.9% and 18.4% higher, respectively [124].

#### 3.1.2. Modeling MBR Systems

The simplicity of the complex process in modeling and simulation is an answer to ‘what ifs’ and this leads to the consideration and varying of factors for optimum response, troubleshooting, and data collection for designing new systems using virtual applications [125]. In terms of simulating and predicting the PW concentration, discharge, dispersion, and environmental risks, four mathematical modeling techniques which are empirical and analytical solutions aimed to develop expressions of the plume parameters; numerical methods for directly solving the advection-diffusion equation on fluxes of pollutants; random walk particle tracking (RWPT) model for tracking individual particle transport; and jet-type integral methods based on the mass, momentum, and concentration and buoyancy conservation are widely used [11,126]. Others include the integration of the Princeton hydrodynamic ocean model and random walk model [127]. Statistical modeling has also been used to achieve similar objectives by using an analytical technique in assessing the PW contaminant levels and its ecological impact [128,129].

Modeling and simulation techniques have also been used in PW treatment, specifically the treatment of PW using MBRs. The Box–Behnken statistical experimental design was used to study the effect of HRT (16–32 h), SRT (60–120 days) and Temperature (22–38 °C) on COD, TOC and oxygen uptake rate (OUR) [130]. Using a hollow fiber submerged UF membrane continuous MBR, Janson et al. [130] reported that the average COD, TOC, and OUR removals were 60%, 59%, and 0.13 mg with high removals occurring at low HRT. Furthermore, no specific trend was observed as it would have been expected that the mixed liquor volatile suspended solids (MLVSS) would increase with increasing HRT. 

A one-way ANOVA acclimatization of the aerobic non-halophilic bacteria of two MBR systems with MLSS adjusted at 5834 ± 877 mg/L and 6655 ± 643 mg/L, respectively was conducted at a varied C/N/P ratio of 100/10/1 and 100/2/1. The reduction in nitrogen in the C/N/P ratio from 10 to 2 inhibited the growth and metabolism of bacteria, thereby causing the reduction in average MLSS for each system. Steady state conditions with statistical significance (*p*-value less than 0.05) was achieved after 21 days [116].

Optimum range of values of the functional dimensions and designed parameters of a submerged CSTR MBR was obtained by simulating the MBR’s performance on COD, TSS, TOC, TDS, and oil and grease removal [131]. The performance equations based on the law of conservation of mass were developed with assumptions such as constant flowrate, no concentration gradient, no contaminant diffusion/dispersion and operation under isothermal, isobaric, and steady state conditions. From Dagde et al.’s [131] model, different volume, height, and hydraulic retention time are required to obtain 95% and 99% conversion with an SRT of 82.7 days. The fundamental fact therefore, still remains that the MBR plant is more complicated in both design and operation and there is greater risk of failure [132].

## 4. Fouling and Fouling Controls in MBRs

The performance and life span of membranes are affected by membrane fouling, which is a major setback in the application of membrane-based technologies in wastewater treatment. Massive global attention has been given to fouling in membrane integrated systems attributable to the deposit of foulants such as colloids, hydrophilic dissolved organic matter, salts, sludge flocs, and suspended particulates. This results in reduced permeate flux, increased feed stream pressure, an increase in operational and maintenance costs, and an increased system downtime especially during the treatment of high strength organic matter [133]. The tendency, behavior, and extent of fouling varying is highly dependent on the nature of foulants, mode of operation, and the physico-chemical interaction occurring between the foulants and the properties of the membrane material. Membrane fouling is inevitable in MBR application in PW treatment due to the heterogeneous nature of PWs. As such, it is possible to have all the classification of fouling with respect to the type of foulants—namely, organic, inorganic, or scaling, particulate and biofouling, occurring in MBRs during PW treatment. During treatment, the significant challenges occurs for high bacteria population of 42 × 10^6^ colony-forming units (cfu)/L [134]. The characteristics of the various fouling types are presented in Table 4 [135].

**Figure 6:**
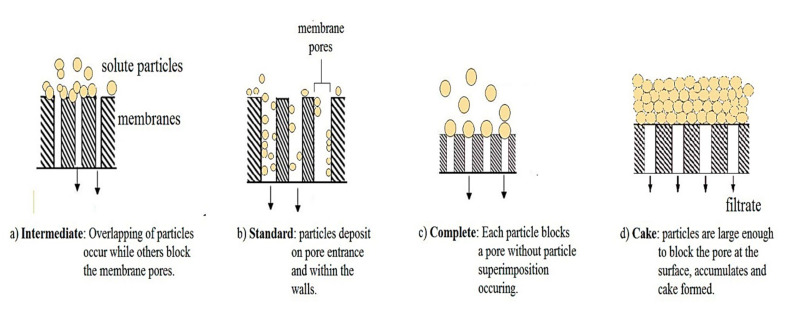
Schematic view of the different pore blocking mechanisms.

Generally, fouling mechanism in MBRs involve adsorption and accumulative deposition of foulants on the surface of the membrane, and precipitation in pore blocking. The surface adsorption, mechanism and accumulative deposition results in reversible fouling while irreversible fouling is attributed to the deposition in the membrane micro pores. Clogging of the pores is dependent on the size and shape of the particle, and the pore and pore size distribution of the membrane. Figure 6 is a schematic view of the different pore blocking mechanisms. The blocking index (*n*) of intermediate blocking, standard blocking, complete blocking, and cake formation are 1, 1.5, 2, and 0, respectively. The rate of fouling is higher in anaerobic MBRs than under aerobic conditions.

### 4.1. Monitoring Fouling

Fouling over the years has been monitored in membrane-based processes through transmembrane pressure (TMP) and change in flux. The change in flux is directly proportional to the change in TMP. In effect, membrane fouling and damaging is reflected through the decline in flux and TMP. 

The propensity of fouling by PW was determined by the flux step method and long-term operation. For PW with an MLSS concentration of 6 g/L, the critical flux was found to be 6 LMH with a corresponding TMP of 12 mbar [114]. Permeability was found to decrease by 50–65% for each 3 LMH flux step between 3–15 LMH and a further decreasing of permeability at an imposed flux of 18 LMH. Similarly, the average flux and TMP of PW with an MLSS of 9.1 g/L increased from 9 LMH and 0.4 kPa to 15 LMH and 2.7 kPa, respectively after 45 days was observed. This prompted the need for a 24 h fouling treatment [111].

The transitional flux for PW with biomass concentration of 6–18 g/L was reportedly between 8–14 LMH. The 6 g/L increments in biomass concentration did not have a direct effect on fouling rates as the fouling rate for high biomass concentration was 36% less than PW with lower concentration [112]. Complexity and variability in the biomass component would be the reason behind the observation, however, PW with high EPS may contribute to severe flux decline due to their large molecular size over membrane pore size [136]. Kose et al. [115] identified the sustainable TMP as 80 kPa and attributed the fouling to physical reversible cake layer and chemical reversible fouling.

### 4.2. General Mitigation Strategies

**Figure 7:**
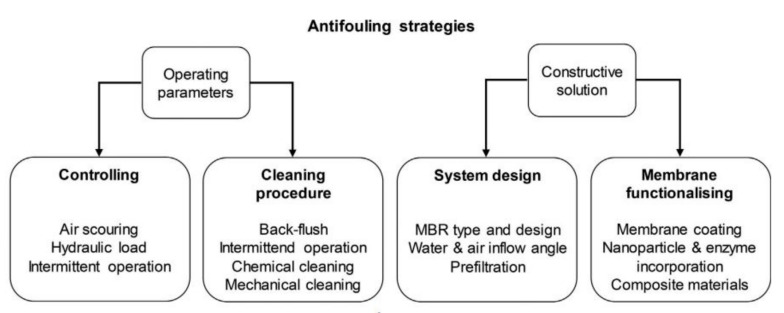
Antifouling strategies adapted from [136].

Strategies to control membrane fouling includes the pretreatment of the feed, optimization of operational conditions, activated sludge modification, membrane design and surface modification, and membrane cleaning. Generally, the antifouling and cleaning activities undertaken to recover the initial permeate flux is determined by the fouling and membrane type used. These strategies are equally major energy enhancing strategies as attempts are made to reduce the overall energy consumption in MBRs. The cost of these activities is also inevitable as membrane permeability maintenance has the most significant impact on operational expenditure [132]. As such, one antifouling technique is the introduction of air flow directly to the membrane surface through additional diffusers below the membrane module. Aeration as a control strategy is a main energy consumer of MBR systems with a percentage of 36–68% [137]. An overview of existing and innovative antifouling strategies for MBRs are illustrated in Figure 7.

Cleaning by ultrasonication has been effective in breaking down thick foulant layers from the membrane surface, however, its effect on anaerobic bacterial activity remains a concern [138,139]. Although research on MBR fouling management in PW treatment is sparse, generic parts such as membrane fouling during MBR wastewater treatment and reclamation [140], membrane fouling from a process control viewpoint [73], and membrane fouling mechanism in anaerobic MBRs [140] exist. Mechanism and limitation of new physical and chemical biofouling control in MBRs are provided in Table 5 [141].

In the area of system design in fouling control, the EMBR, IEMBR, EDIMB, and OsMBR novel systems provide low fouling, high treatment performance, and energy saving characteristics. A number of MBR systems use rotation, vibration (longitudinally, transversely, torsionally, magnetically, or the combination) movement to increase shear-enhanced filtration. To improve cleaning efficiency, eliminate membrane fouling, and stabilize transmembrane pressure, the membrane cassette can also be reciprocated [142,143,144]. Alternative designs have employed the usage of baffles to divide the bioreactor compartment into zones in creating an anoxic–aerobic condition in the reactor vessel for efficient nitrogen removal through simultaneous nitrification and denitrification [145,146].

**Figure 8:**
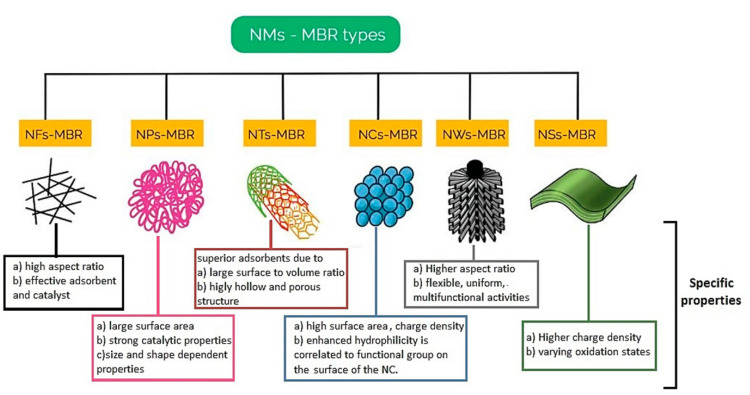
Types of nanomaterials of hybrid NMs-MBR systems adapted and modified from [146,147].

Current practices in improving membrane functionality, high flux and mitigating membrane fouling in MBRs is by embedding nanomaterials into the polymer support structure or deposition on the surface of the membrane to achieve such characteristics such as hydrophilicity, hydraulic stability, antimicrobial ability (inhibition of metabolism), thermal stability, and photocatalytic self-cleaning. The different nanomaterial membrane bioreactors (NMsMBRs) therefore classified by the nanomaterials as nanofibers membrane bioreactor (NFs-MBR), nanotubes membrane bioreactor (NTs-MBR), nano particle membrane bioreactor (NPs-MBR), nanosheets membrane bioreactor (NSs-MBR), nanowires membrane bioreactor (NWs-MBR) and nanocrystals membrane bioreactor (NCs-MBR) [147,148] as illustrated in Figure 8.

A thorough search on the use of NMs-MBR systems for PW treatment revealed recent work by Fonouni et al. [149] and Etemadi et al. [150] by comparing a commercial PP membrane and TiO_2_/PP. While achieving a nanocomposite membrane with porosity and tensile strength of 19.26% and 0.6 MPa higher than commercial PP, respectively. The nanocomposite NM-MBR had higher flux recovery ratio (FRR) and lower irreversible fouling ratio (IFR) to demonstrate its better flux recovery and total fouling control over commercial PP membranes. The analysis of the impact of aeration on fouling, using the Hermia’s model, predicted that at a lower aeration rate, the fouling mechanism was by cake formation [150].

These NMs-MBRs have shown various membrane functional limitations and a few generalized to specific drawbacks are listed below:

(1) NFs, such as Ag/polyamine, decay as a result of irreversible fouling [151,152].
(2) Recovery of NP to encourage reusability concerns, accumulation of NP in MBR and the leaching of NPs. Additionally, the stability of TiO_2_ on PES, PVDF, and PAN in ensuring fouling mitigation is affected by the immobilization technique [153,154].
(3) Be it single walled carbon nanotubes (SWCNT) or multiwalled carbon nanotubes (MWCNT), graphene oxide or reduced graphene oxide application in NTs-MBR, the functionality and properties are specific to the surface modification technique. This mostly contributes to specific affinity and long term stability is not assured due to poor dispersion over time [155,156].
(4) Pristine cellulose NC-MBR and modified cellulose NCs have limited membrane lifetime due to biodegradability of cellulose [157].

## 5. Future Perspective

In spite of the progress in the usage of MBRs in treating different wastewater and the continuous evolution of membrane technology, there are limited applications of MBR technology in PW treatment. There are challenges to be addressed from a laboratory scale before an acceleration into large-commercial scale application.

- Very useful data is available from peer-reviewed literature on the treatment of PW using biological and membrane technology. However, the use of MBR systems (including hybrid structures) and its integration with other treatment systems such as RO, NMs (NPs-MBR, NTs-MBR, NCs-MBR, NWs-MBR, and NSs-MBR) and AOP is limited, and much focus must be channeled to establish the independent process efficiency and synergic output.
- With PW being comprised of more different components than just oil–water emulsion, the individual interactive influence of PW components on properties and parameters of a conventional MBR and modified system can be studied systematically to give new insights. For instance, the degradation chemistry of initial pollutants should be understood. Additionally, dynamic models could be developed which should focus on individual characteristic treatment and hydrodynamic flow behavior of synthetic and real feeds in the reactor.
- The impact of chemical, physical, biological fouling, and constructive control strategies on the performance of MBRs on laboratory and pilot scale must be conducted in relation to duration, dosage, metabolic activity, process stability, membrane improvement, behavior in active layer transport of membrane and sustainability, effectiveness, and environmental safety. The development of modified multi-functional low-cost membranes with superior antifouling characteristics can be pursued.
- The high salinity and hydrocarbon content of PW makes PW treatment very energy demanding. Owing to on-site MBR systems, another research direction focusing on powering MBR systems with renewable energies coupled with intelligent process monitoring control systems in achieving an autonomous MBR system should be carried out.

## 6. Conclusions

The implementation of ecofriendly wastewater technologies is important in achieving management policies of meeting discharge limits and increasing reusability of treated wastewater as a source water. MBR technology offers process advantages over conventional activated sludge processes, adsorption technology, hydrocyclones, gravity settling, precipitation, and many more. This article reviews the treatment of PW using membrane bioreactors which included the treatment schemes, models, and integrated processes. Other aspects addressed includes general fouling and fouling control associated with MBRs for PW treatment. The use of MBRs have demonstrated good performances in the removal of pollutants; however, there are several research gaps to be filled in that area. The scopes should include the MBR configuration and hybrid systems for improved treatment. The cost of large-scale manufacturing of membranes, a key component of MBR and the chemistry of degradation of pollutants in conventional and control of fouling using modified membranes should also be considered. The continuous advancements, especially in membrane design and technology, is key in achieving a process and energy efficient MBR for PW treatment.

## Figures and Tables

**Table 1:** Produced water composition.

Category	Type	Comments	References
Suspended solids	Formation solids, sand, silt, carbonate, bacteria, waxes, asphaltenes, scale, and corrosion products	High molecular weight PAH are sorbed onto suspended solids.	[45,46]
Petroleum hydrocarbons (dissolved and dispersed oils)	Aliphatic hydrocarbons, BTEX phenols, carboxylic acid, mono aromatic hydrocarbons (MAH), dispersed poly aromatic hydrocarbons (PAH)	Aliphatic hydrocarbons, phenols, low molecular weight PAH are soluble. PAH such as pyrene is mutagenic while benz[*a*]anthracene and benzo[*a*]pyrene are considered moderate-to-weak and potent carcinogens, respectively.	[47,48,49,50,51]
Heavy metals	Iron, cadmium, chromium, zinc, lead, strontium, mercury, nickel, silver, barium, copper, cobalt, selenium	Found in trace concentration. Exposure to these heavy metals causes central nervous system disorder, fertility problems, sinus node dysfunction, liver necrosis, rheumatoid arthritis, and cardiovascular diseases. Adverse effect on aquatic life	[52,53,54,55]
Bacteria	Bacillales, Halanaerobiales, Halanaerobrium, Fusobacteria, Pseudomonadales,	Potential for souring, causes corrosion and fouling of pipelines, biogenic gases. PW with sulfate reducing bacteria count between 100–1000/mL requires treatment.	[56,57,58]
TERNOM	Radium (^224^Ra, ^226^Ra and ^228^Ra), uranium (^238^U), thorium (^232^Th).	Radium isotopes are mostly present and decay into radon (Rn^232^). Continuous exposure leads to bone and sinus cancer. Accumulated solids with NORM are mostly cleaned out and disposed in a controlled process.	[59,60,61]
Inorganic salts	CaCl_2_, MgCl_2_, and NaCl	Affects conductivity, clogs pipes on accumulation and can cause severe soil erosion.	[39,62]
Dissolved gasses	CO_2_, O_2_, H_2_S, N_2_	Dissolved gases also include the alkane gases which is mostly dominated by methane. H_2_S is highly toxic and corrosive. CO_2_ is corrosive and results in CaCO_3_ scaling.	[63,64]

**Table 2:** Advantages and disadvantages of selected PW treatment technologies [57,75,76,77,78,79,80,81].

Category	Technology	Advantage	Disadvantages
Physical	Evaporation	Eliminates chemical application and physical treatment, no chemical sludge produced, less maintenance involved.	High energy cost, concentrated, brine sludge might require secondary treatment prior to disposal.
	Adsorption (zeolites, activated carbon, activated alumina, organoclays)	Simple technology, low-cost materials, low energy requirement. High heavy metals efficiency for soluble BTEX (benzene, toulene, ethyl benzene, xylene) and insoluble free hydrocarbons.	Chemical sludge generation, plugging of sorbent active sites by organics, frequent regeneration of adsorbents. Performance of absorbent is a function of temperature, pH, suspended solids, salinity, and dissolved organic contaminants.
	Gravity Settling (skim vessels, API tanks and parallel and corrugated plate separators-PCPS)	Simple equipment, high separation for large oil droplets (>150 μm, PCPS- 40 μm), minimum operational and maintenance cost, >60% free water removal.	Large footprint, ineffective on dissolved contaminants, longer settling time for smaller droplets, PCPS not suitable for heavy oil separation and also susceptible to plate clogging.
	Hydrocyclones (static and dynamic)	Easily accessible, compact in design with low retention time, low capital cost and low maintenance cost, functions best as a pre-treatment device, high oil/water separation >75% with droplet size above 50 μm.	Generally low contaminant removal efficiency, oil/water separation is affected by oil droplet size (minimum = 10–15 microns), pressure drop ratio and inlet solid concentration, high maintenance, and does not remove dissolved components, high susceptibility to blockages and fouling, higher pressure drops, pump required for oils are low pressure which can also reduce oil droplet size
	Gas Floatation (Dissolved gas, dispersed gas and hydraulic induced)	Simplicity of design and operation compared to gravity settlers, high oil recovery (>80%) for inlet oil concentration between 250–500 mg/L, effective removal of less dense particles, low to moderate energy demand, overall footprint can be small, hydraulic induced units capable of operating above atmospheric pressure.	Scaling of units when PW has high dissolved solid content, pressure, and liquid level control is required; surfactants, flocculants, and demulsifiers; chemical requirement increases cost of treatment; bubble size decreases with increasing salinity.
Chemical	Precipitation	High removal (<90%) for insoluble contaminants, removal of large oil droplets, solid and organic carbons	High chemical demand, large sludge production, sludge matrix consists of precipitant, not effective for dissolved contaminants, hydrophilic compounds and nitrogen.
	Oxidation (advanced process)	Can achieve 100% water recovery rate, smaller footprint, high degradation rate (>70%), minimum to no solid residual production, photocatalysis has lower TOC removal (<20%)	High chemical cost and production of unknown transformational products. Complex system that requires skilled operators.

**Table 3:** Performance of MBR in produced water treatment.

MBR					Influent		Effluent	Ref.
Membrane			System	Operational Conditions				
Material	Brand	Model			Feed Type	Composition		
Chlorinated PE	Commercial	Flat sheet	SMBR anoxic-aeration system	HRT = 13–19 h, SRT = 600 h, Flux = 9–15 LMH, DO = 0.3–4 mg/L	Real	pH = 6.4–10.4, COD = 720–159 mg/L, PO_4_ = 8.5–10.1 mg/L, NH_4_-N = 56–132 mg/L, Oil and grease = 14–20 mg/L	pH = 7.6–8.6, COD = < 5.2%, PO_4_ = < 35%, NH_4_-N = < 0.72%, oil and grease = < 20%	[111]
PVDF	Commercial	Tubular asymmetric	SMBR and hybrid with airlift	HRT = 12–24 h, SRT = 720 h, Flux = 8–18 LMH, DO > 2.5 mg/L	synthetic	COD = 1575–2000 mg/L, Benzene = 30–70 mg/L, Toulene = 19–40 mg/L, Ethylbenzene = 4–8 mg/L, Xylene = 10–20 mg/L	COD = < 10%, TOC = < 3%, BTEX = < 1%	[112]
PVDF	Commercial	Hollow fiber	SMBR	HRT = 24 h, SRT = 20 days,	synthetic	TSS = 985–1381 mg/L, VSS = 937–1213 mg/L, COD = 353–427 mg/L	TSS = < 23% and VSS = < 26%, COD = < 9%	[113]
-	Commercial	Tubular	SMBR	HRT = 24 h, SRT = 30 days, Flow: 0.125 L/h	synthetic	COD = 1475–1575 mg/L, BTEX = 4000–35,000 ug/L,	COD = < 0.5%, BTEX = < 0.2%	[114]
PP	Commercial	Hollow fiber	Continuous flow SMBR	HRT/SRT = 30–250 days, Flux = 10 LMH, F/M ratio: 0.25–0.55	Real	Oil and grease = 31–47 mg/L, TPH = 1030–2210 ppm, COD = 1500–3000 mg/L	Oil and grease = < 31%, TPH = < 6%, COD = < 21%	[115]
PVDF	Commercial	Flat sheet	SMBR with Homogenizer	HRT = 2.67 Days, SRT = 80 days, DO = 3 mg/L, Flux = 1.99 LMH, OLR = 0.975 gCOD L^−1^ d^−1^	Synthetic	COD = < 2600 mg/L, Oil and grease = 1750 mg/L	COD = < 10.07%, Oil and grease = < 4.04%, NH_3_N = 6.55%, PO_4_^3−^ = 38–53.51%	[116]

COD = Chemical oxygen demand; DO = Dissolved oxygen; HR = Hydraulic retention time; TPH = Total petroleum hydrocarbon; TP = Total phosphorus; TSS = Total suspended solids; SRT = Solid retention time; OLR = Organic loading rate; VSS = Volatile suspended solids.

**Table 4:** Different foulants and their characteristics [135].

Characteristics	Fouling Type			
	Particulate	Biofouling	Inorganic	Organic
Foulants	Suspended solids	Extracellular polymeric substances (EPS)	Mineral salts, metal cations	NOM, proteins, polysaccharides, fatty acids
Affecting Factors	Concentration, charge, shape, ion interaction, size, compressibility	Temperature and nutrients	Concentration, temperature, and pH	pH, concentration, hydrophobicity, ionic strength
Prediction Indicators	Modified fouling index, specific fouling resistance, silt density index	Assimilable organic carbon, rate of biofilm formation	Solubility	Specific ultraviolet adsorption, DOC, ultraviolet254
Mechanism	Organic and inorganic fouling mechanism	Induction accumulation, logarithmical growth, biofilm layer	Crystallization on membrane surface	Pore blocking and cake formation

**Table 5:** Mechanism and limitation of selection physical and chemical biofouling control processes.

Method	Mechanism	Limitation
Ultrasonic cleaning	Shear force, drag force, difference in pressure and high-pressure shock wave, agglomeration of small particles	Decompose sludge into small particles, increases extracellular polymeric substance adhesion, membrane damage
Electric field assistance	Deposition of sludge and colloids on the membrane surface are prevented; promote the metabolism in microorganism; H_2_O oxidization	Complex operational process, high cost
(Chemical) Ferric oxide, Peroxymonosulfate	Biomass floc size is increased, Enhancement of microorganism activity, Oxidize and degrade dirt	limited ability of the chemical process to remove membrane biofouling, High chemical demand, High cost of chemicals. Associated ecological, environmental, and high risk
Ozone	Mainly expands the sludge flocs by reducing the zeta potential value, surface hydrophobicity of floc increases	-

## Data Availability

Not applicable.
